# Vagus nerve stimulation modulates synaptic plasticity induced by cocaine- seeking in reward-related circuitry

**DOI:** 10.21203/rs.3.rs-7014180/v1

**Published:** 2025-07-16

**Authors:** Reza Arezoomandan, Lily Vu, Christopher Driskill, Sven Kroener

**Affiliations:** The University of Texas at Dallas; The University of Texas at Dallas; The University of Texas at Dallas; The University of Texas at Dallas

**Keywords:** Cocaine, Vagus nerve stimulation, Local field potentials, LTP, Prefrontal cortex

## Abstract

Cocaine use alters brain networks and connections, impairing inhibitory control over drug-seeking. Cortical-limbic circuits, including the infralimbic (IL), prelimbic (PL) cortices, and basolateral amygdala (BLA), regulate extinction learning and drug-seeking via projections to the nucleus accumbens (NAc). Vagus nerve stimulation (VNS) paired with extinction enhances learning and reduces reinstatement, but its effects on extinction-related networks remain unclear. This study examined how cocaine and VNS affect plasticity in relapse-related pathways. Evoked local field potentials (eLFP) were recorded in the IL, NAc core, and NAc shell following self-administration or reinstatement sessions. In the BLA-IL pathway, cocaine-treated (COC) and sham-VNS (SHAM) groups exhibited the highest baseline eLFP amplitudes and increased long-term potentiation (LTP) induction, which VNS restored to yoked-saline (YS) levels. In the PL-NAc core pathway, high-frequency stimulation (HFS) had no effect on EFPs in VNS-treated animals, significantly differing from the long-term depression (LTD) observed in COC and SHAM groups, which had the highest baseline eLFP amplitudes. In the IL-NAc shell pathway, VNS-treated rats displayed the largest baseline amplitudes, and unlike YS, COC, and SHAM groups, HFS in the IL induced persistent LTP in the NAc shell. These findings suggest cocaine use and craving induce maladaptive neuroplasticity within cortical-limbic circuits, and VNS may modulate these changes, contributing its beneficial effects in preventing reinstatement.

## Introduction

Cocaine addiction is a chronic and relapsing disorder characterized by maladaptive neuroplasticity within cortical-limbic circuits. These circuits, which include the prefrontal cortex (PFC), nucleus accumbens (NAc), and amygdala, are critical for regulating reward processing, emotional responses, and decision-making ^1^. Chronic cocaine exposure disrupts the balance of synaptic plasticity in these pathways, including impairment in induction of LTP and LTD, resulting in heightened sensitivity to drug-related cues and impaired behavioral control ^1–3^. These changes underlie the persistent drug-seeking behaviors and high relapse rates observed in cocaine addiction ^1^. The infralimbic cortex (IL) and prelimbic cortex (PL), two subregions of the PFC, play largely opposing roles in drug-seeking behavior. The IL inhibits drug-seeking by projecting to the NAc shell or PL, while the PL drives drug-seeking and facilitates reinstatement of drug-seeking through its connections to the NAc core ^4^. The basolateral amygdala (BLA) is another key structure in this network, encoding the salience of drug-related cues and influencing the activity of other brain areas involved in drug-seeking including PFC and NAc ^5–7^.

Chronic cocaine use induces significant molecular and cellular changes in reward-related brain regions, leading to disruptions in normal brain function, including impairments in synaptic plasticity ^1^. Cocaine induces neuroadaptations within cortical-limbic pathways, which is strongly implicated in the persistence of addictive behaviors. Cocaine induces metaplasticity, a phenomenon linked to increased vulnerability to relapse ^2,8^. Metaplasticity, defined as a change in threshold or rule for inducing synaptic plasticity, represents a higher-order form of plasticity that is not expressed as a change in synaptic transmission efficacy but rather as a change in the direction or degree of plasticity induced by specific stimulation patterns ^9^. This concept is essential for understanding drug addiction mechanisms. Effective treatments for relapse should normalize synaptic transmission and reverse cocaine-induced metaplasticity in reward-related brain areas.

Vagus nerve stimulation (VNS) is FDA-approved for use in epilepsy and depression and considered for the treatment of an expanding range of psychiatric diseases including substance use disorders ^10^. VNS causes the release of several neuromodulators which modulate cortical plasticity. This plasticity can increase learning and memory in rats ^11,12^ and humans ^13^. Recently we showed that VNS facilitates extinction and reduces cue‐induced reinstatement in cocaine self‐administering rats ^14–16^. Also, we have shown that pairing VNS with extinction of conditioned fear potentiates evoked field responses in the BLA, resulting in LTP in the IL-BLA pathway, suggesting that VNS promotes plasticity in this pathway to facilitate extinction of conditioned fear responses ^17^. VNS has been shown to reduce relapse in reinstatement models of drug-seeking as well as modulate synaptic transmission and metaplasticity ^14–19^.

Here we determined how cocaine self-administration and reinstatement induce metaplasticity in keycortical-limbic circuits involved in drug-seeking, including the BLA-IL, IL-NAc shell and PL-NAc corepathways. We also show that extinction training paired with VNS can modulate these changes to reducereinstatement.

## Results

### VNS facilitates extinction and reduces cue-induced reinstatement

**Figure 1:**
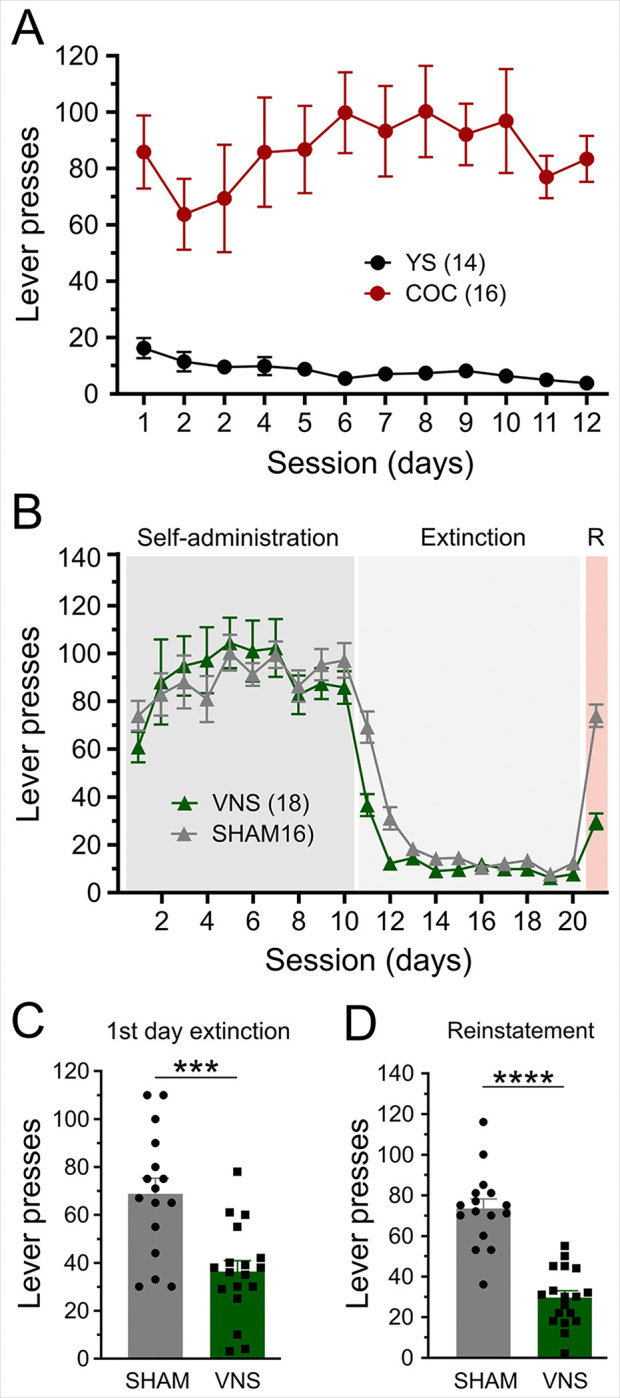
Vagus nerve stimulation (VNS) facilitates extinction from cocaine seeking and reduces cue-induced reinstatement. (A) Active lever presses in rats self-administering cocaine (COC, red circles) and their yoked-saline controls (YS, black circles) during the last 10 days of self-administration. (B) Responses at the active lever during self-administration, extinction, and cue-induced reinstatement in rats receiving VNS (green triangles) or sham stimulation (SHAM, gray triangles) during extinction on days 11–20. (C) VNS-treated rats displayed reduced active lever presses during the first day of extinction. (D) An unpaired t-test revealed significantly fewer responses on the previously active lever during cue-induced reinstatement in VNS rats. *P* values are (***) <0.001, and (****) <0.0001.

To examine the effects of cocaine self-administration on neuroplasticity, 30 rats of both sexes (16 males; 14 females) were trained to self-administer either cocaine (COC, n = 16) or which received yoked-saline infusions (YS, n = 14) for at least 10 days. Figure 1A shows active lever presses in a cohort of COS and YS rats. Cocaine self-administering animals consistently exhibited significantly higher active lever responses compared to yoked-saline animals (F(1, 28) = 46.56, *P* < 0.0001). To evaluate the impact of VNS on drug-seeking behavior, a separate group of rats underwent cocaine self-administration for a minimum of 10 days, followed by 10 days of extinction training paired with either VNS (n = 18, 10 males and 8 females) or sham stimulation (SHAM; n = 16, 9 males and 7 females). After 10 days of extinction, drug-seeking behavior was assessed in a cued reinstatement session through the presentation of conditioned drug cues (Fig. 1B). A two-way ANOVA revealed no significant differences between groups in active lever responses during the final 10 days of self-administration (F (1, 33) = 0.013, *P* = 0.9). During extinction training and the cue-induced reinstatement session, responses at the previously active lever were used as a measure of extinction learning. A repeated-measures two-way ANOVA, with time (sessions) and treatment (SHAM or VNS) as factors, revealed a significant effect of extinction (main effect of time: F (9, 330) = 57.67, *P* < 0.0001), a significant difference between treatment groups (main effect of treatment: F (1, 330) = 46.59, p < 0.0001), and a significant interaction between these factors (F (9, 330) = 8.52, *P* < 0.0001; Fig. 1B). Post hoc analysis showed that rats receiving VNS pressed the active lever significantly less than the sham group on the first two days of extinction (Day 1, *P* < 0.0001, Fig. 1B; Day 2, *P* < 0.0001, Fig. 1B, C), suggesting that VNS facilitates extinction learning. Twenty-four hours after the final extinction session, drug-seeking was assessed in a cue-induced reinstatement session. An unpaired t-test revealed that VNS animals exhibited significantly fewer responses on the previously active lever during cue-induced reinstatement (t(32) = 7.79, *P* < 0.0001; Fig. 1B, D), demonstrating the effectiveness of VNS in inhibiting cue-induced reinstatement.

### VNS reverses drug-induced LTP in the pathway from the BLA to the IL

**Figure 2:**
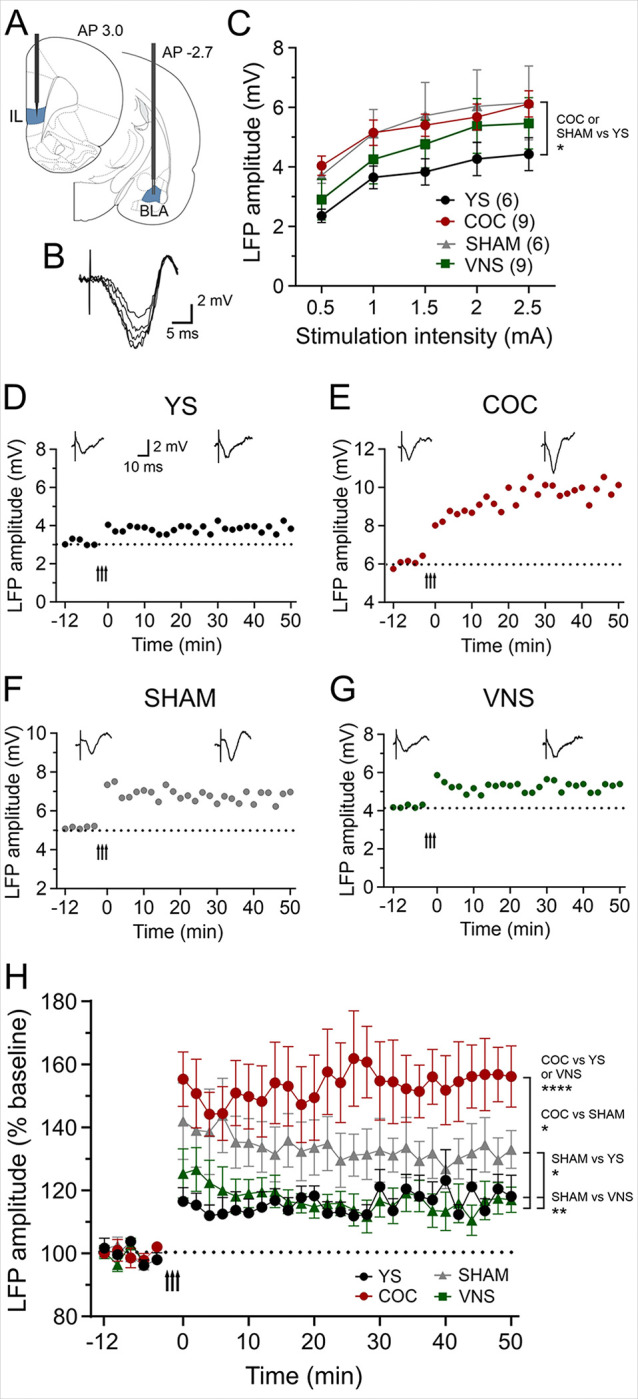
VNS reverses drug-induced LTP in the BLA-IL pathway. (A) Diagram of stimulation and recording sites in the IL and BLA. (B) Single-pulse stimulation in the BLA elicited negative field potentials in the IL that peaked after ~10 ms. Representative traces (averages of ten consecutive sweeps) of an input–output curve from a YS rat are shown. (C) Input–output curves in the four treatment groups. Cocaine self-administering (COC) and sham-stimulated (SHAM) groups showed increased baseline responses relative to eLFPs in yoked-saline (YS). VNS treatment modified changes induced by drug-seeking and decreased eLFP amplitudes. (D-G) Representative experiments showing eLFPs in all four treatment groups before and after HFS (50 Hz; arrow indicates delivery). Insets are averages of eight consecutive eLFPs before and after HFS. (H) Comparison of synaptic plasticity changes across the four treatment groups. HFS induced LTP in all groups, with the greatest change in COC and SHAM rats (*P*< 0.0001 and *P*= 0.024, respectively). Extinction paired with VNS reversed drug-seeking-induced LTP, restoring it to YS control levels (*P*= 0.004, VNS vs SHAM). BLA: basolateral amygdala, IL: infralimbic cortex.

To determine how cocaine self-administration, reinstatement, and VNS influence synaptic plasticity in the pathway from the BLA to the IL, we performed *in vivo* recordings in anesthetized rats following the final session of either self-administration or reinstatement in the four treatment groups (YS, n = 6; COC, n = 9; SHAM, n = 6; VNS, n = 9). We placed a stimulation electrode into the BLA and performed recordings of eLFPs in the IL (Fig. 2A). Single-pulse stimulation in the BLA evoked a characteristic field potential response in the IL which peaked after 10ms (Fig. 2B). A two-way ANOVA examining the current-voltage relationship of eLFPs revealed a main effect of I–O curves (F (3, 140) = 7.185; *P* = 0.0002, Fig. 2C). In the BLA-IL pathway, cocaine self-administration and cue-induced reinstatement significantly altered the current-voltage relationship of eLFPs, resulting in a marked increase in baseline responses relative to eLFPs in YS rats (*P* < 0.05). VNS treatment reversed these changes and reduced baseline responses, resulting in no significant difference between the VNS and YS groups (Fig. 2C). After obtaining stable baseline recordings for a minimum of 10 minutes we used HFS in the BLA to induce LTP to further examine the effect of drug-seeking and VNS treatment on synaptic plasticity in this pathway. Tetanic stimulation delivered to the BLA induced a persistent increase in amplitude of BLA-evoked responses in the IL in all groups (Fig. 2D– H); however, the magnitude of LTP was significantly greater in COC rats compared to LTP in the other groups F (3, 50) = 6.827; *P* = 0.0006, Fig. 2E, H). SHAM animals exhibited smaller LTP compared to rats in the COC group (*P* = 0.021; Fig. 2F, H), but larger amplitudes than rats in the YS group (*P* = 0.024; 2H). Pairing extinction training with VNS significantly reversed the drug-seeking-induced LTP, restoring it to YS control levels (Fig. 2G, H). These results show that drug-seeking behavior strengthens this pathway, and that extinction training by itself can partially reverse these changes. Pairing extinction training with VNS further reduces drug-induced changes resulting in plasticity similar to that observed in YS controls.

### VNS modulates drug-induced changes in synaptic plasticity in the PL-NAc core pathway

**Figure 3:**
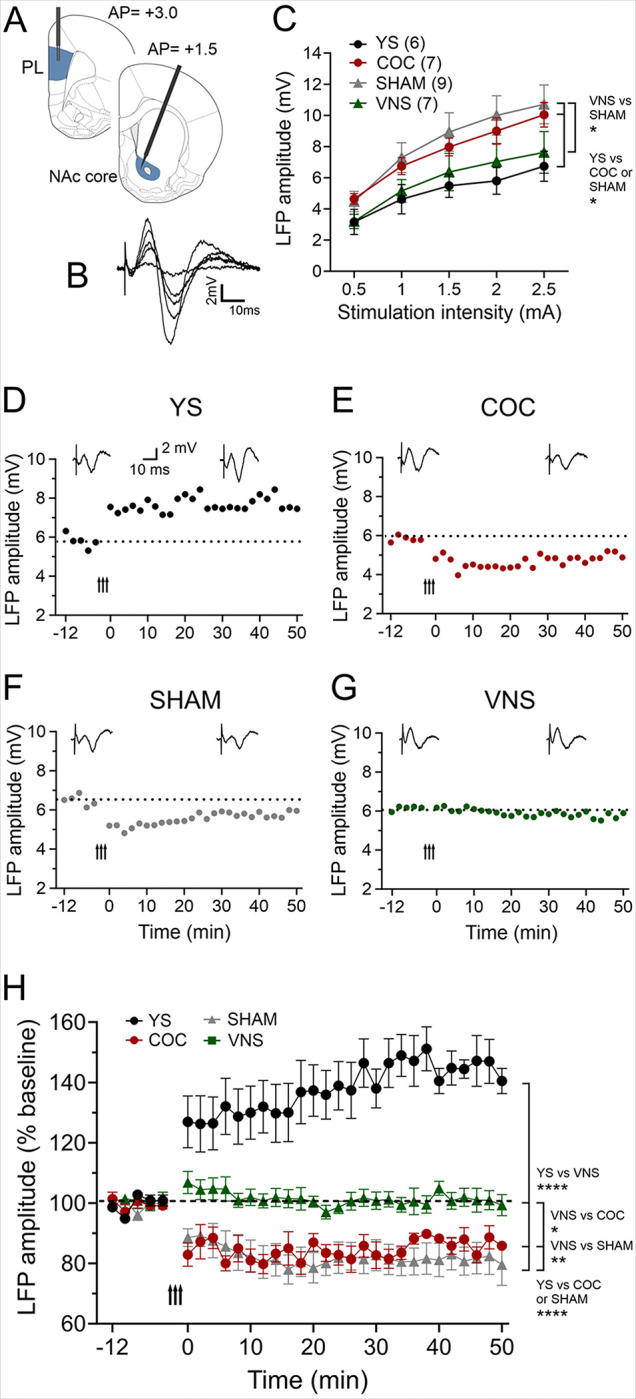
VNS modulates drug-induced changes in synaptic plasticity in the PL-NAc core pathway. (A) Diagram of stimulation and recording sites in the PL and NAc core. (B) Single-pulse stimulation in the PL elicited negative field potentials in the NAc core peaking at 20–25 ms. Representative traces (average of ten consecutive sweeps) from a yoked-saline (YS) rat. (C) Input–output curves from rats in four treatment groups. Cocaine self-administration (COC) and cue-induced reinstatement (SHAM) increased baseline responses compared to YS rats (*P* <0.05). VNS treatment partially reversed this effect, resulting in smaller amplitudes than in COC and SHAM rats (*P* <0.05). (D–G) Representative experiments showing plasticity induced by HFS (50 Hz; arrow indicates delivery) in the four treatment groups. Insets show the average of eight consecutive eLFPs before and after HFS. (H) Comparison of average plasticity changes across the four treatment groups. HFS induced LTP in YS rats (*P*<0.0001) but caused LTD in COC and SHAM rats (*P*= 0.012 and *P*= 0.0005, respectively). In VNS-treated rats, HFS failed to induce either LTP or LTD which was significantly different from the LTD induced in COC and SHAM-treated rats (*P*= 0.025 and *P*= 0.001, respectively). PL: prelimbic cortex, NAc: nucleus accumbens.

The PL can drive reinstatement of drug-seeking via its projection to the NAc core ^4^. To determine how cocaine self-administration and cue-induced reinstatement alter synaptic plasticity in the PL-NAc core pathway, and how pairing extinction training with VNS can modulate these changes, we recorded eLFPs in this pathway in our four treatment groups (YS, n = 6; COC, n = 7; SHAM, n = 9; VNS, n = 9) after the last self-administration session or the reinstatement session, respectively (Fig. 1A, B, 3A). Stimulation of the PL elicited negative field potentials in the NAc core, which peaked after 20–25ms (Fig. 3B). The current-voltage relationship between stimulation intensity in the PL and eLFP amplitude in the NAc core were significantly different between the four groups F (3, 135) = 6.509; *P* = 0.0004; Fig. 3C). The COC and SHAM groups showed increased baseline eLFP amplitudes compared to the YS group (*P* < 0.05). In addition, eLFP amplitudes in the VNS group were significantly reduced compared to those in COC and SHAM rats (*P* < 0.05). (Fig. 3C). To further examine the effect of drug-seeking and VNS treatment on synaptic plasticity we used HFS (50 Hz) of the PL to induce LTP in this pathway. Our results showed a main effect of treatment F (3, 56) = 26.04, *P* < 0.0001). Consistent with previous reports ^2^, this protocol induced synaptic plasticity in YS rats (*P* < 0.0001, Fig. 3D, H). In contrast, in COC and SHAM animals, HFS consistently induced LTD rather than LTP in this pathway (*P* = 0.012 and *P* = 0.0005, respectively, Fig. 3E, F, H). In VNS rats, HFS did not induce synaptic plasticity (baseline vs. post HFS, *P* = 0.9), which was significantly different from the LTD induced in COC and SHAM-treated rats (*P* = 0.025 and *P* = 0.001, respectively, Fig. 3F, H). These results show that VNS can modulate drug-seeking induced changes in metaplasticity in the PL-NAc core pathway.

### VNS Induces synaptic plasticity in the IL-NAc shell pathway

**Figure 4:**
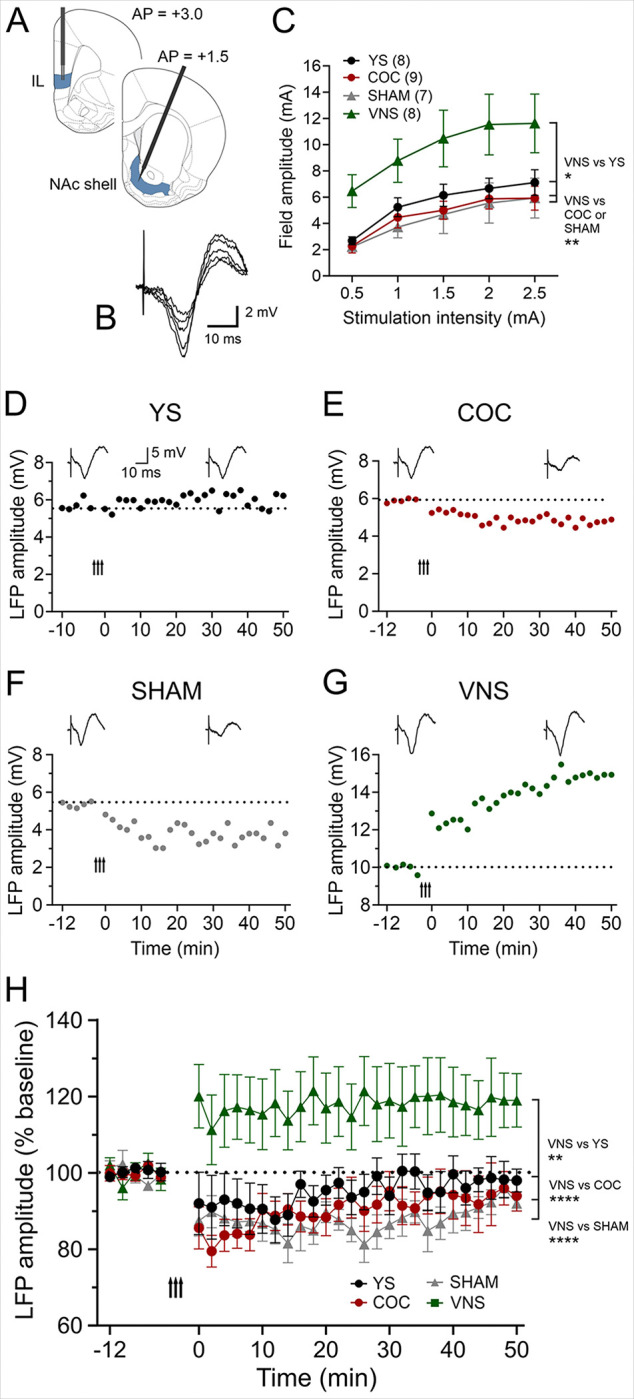
VNS induces synaptic plasticity in the IL-NAc shell pathway. (A) Diagram of stimulation and recording sites in the IL and NAc shell. (B) Single-pulse stimulation targeted to the IL elicited negative field potentials in the NAc shell peaking at ~10 ms. Representative traces (average of ten consecutive sweeps) from a YS rat. (C) Input–output curves from rats in the four treatment groups. VNS treatment significantly enhanced baseline amplitudes (*P*< 0.01, VNS vs YS). (D–G) Representative experiments showing plasticity induced by HFS (50 Hz; arrow indicates delivery) in the four treatment groups. Insets show the average of eight consecutive eLFPs before and after HFS. (D) HFS failed to induce LTP in YS rats. (E, F) In COC and SHAM rats, HFS induced LTD instead of LTP (*P*= 0.043 and *P*= 0.042, respectively). (G) In VNS-treated rats, HFS successfully induced LTP in the NAc shell compared to both the baseline and SHAM group (*P*= 0.001 and *P*<0.0001 respectively). (H) Group comparisons of plasticity outcomes. COC use and reinstatement shifted IL–NAc plasticity toward LTD, whereas VNS paired with extinction changed this pathway to induction of LTP. IL: infralimbic, NAc: nucleus accumbens.

The IL facilitates extinction learning and suppresses cocaine-seeking behavior through its projections to the NAc shell ^20^. To determine how cocaine self-administration, reinstatement, and VNS influence synaptic plasticity we recorded eLFPs in this pathway in the four treatment groups after the last self-administration or the reinstatement session (YS, n = 8; COC, n = 9) or reinstatement (SHAM, n = 7; VNS, n = 8) (Fig. 4A). IL stimulation elicited eLFPs in the NAc shell, characterized by a negative potential peaking at 10ms (Fig. 4B). The shape, amplitude, and latency of these eLFPs were highly reproducible and remained stable over time. A two-way ANOVA assessing the relationship between stimulation intensity (0.2 mA–2.5 mA) and baseline eLFP amplitude revealed a significant main effect of treatment on I–O curves (F(3,144) = 19.91, *P* < 0.0001). Specifically, rats in the VNS group exhibited significantly larger baseline responses compared to YS (P < 0.05), as well as COC and SHAM rats (P < 0.01, Fig. 4C). After obtaining stable baseline recordings, we applied HFS to induce LTP. In YS rats, HFS of the IL failed to induce synaptic plasticity in the NAc shell, showing no significant difference between baseline and post-HFS amplitudes (*P* = 0. 0.88, Fig. 2D, H). In both COC and SHAM rats, post-HFS amplitudes remained consistently lower, indicating that cocaine self-administration and reinstatement facilitated induction of LTD rather than LTP in this pathway (*P* = 0.043 and *P* = 0.042 respectively, Fig. 2E, F, H). In contrast, pairing extinction training with VNS modified metaplasticity in the NAc shell so that HFS of the IL induced LTP in the NAc shell compared to both the baseline and SHAM group (*P* = 0.001 and *P* < 0.0001 respectively).Taken together, these findings demonstrate that both cocaine-seeking behavior and VNS significantly alter synaptic plasticity in the IL-to-NAc shell pathway (F (3, 56) = 4.475, *P =* 0.0069). While drug-seeking behavior weakens this pathway, pairing extinction training with VNS strengthens it.

## Discussion

Corticolimbic pathways connecting the PFC, amygdala, and NAc play a crucial role in drug-seeking behavior by integrating emotional cues, reward anticipation, and decision-making ^21^. Dysregulation in these pathways reinforces maladaptive behaviors and impairs inhibitory control, perpetuating addiction ^1,2^. While extinction training can reverse drug-induced changes, it alone is often insufficient to prevent reinstatement. We replicate previous findings showing that pairing extinction training with VNS accelerates extinction learning during the initial session and reduces cue-induced reinstatement, suggesting enhanced consolidation of extinction memories ^14,16^. We determined changes in synaptic metaplasticity induced by drug-taking and cue-induced reinstatement in key cortical-limbic circuits, including the BLA-IL, IL-NAc shell, and PL-NAc core pathways. We show that drug-seeking behavior disrupts synaptic plasticity in each of these circuits and that pairing extinction with VNS effectively reverses these neuroplastic changes in ways consistent with VNS-induced suppression of drug-seeking.

The BLA mediates the consolidation of both cocaine-stimulus association and extinction learning, two processes with opposite effects on subsequent cue-induced cocaine-seeking behavior ^5^. The BLA processes the salience of drug-related stimuli within the corticolimbic circuit ^6^, contributing to cue-induced reinstatement of cocaine-seeking ^22,23^. Inactivation of the BLA, along with dorsomedial PFC inactivation, inhibits conditioned cue-induced reinstatement of cocaine-seeking behavior ^6^, while cocaine-associated cues trigger c-Fos activation in BLA-to-mPFC pathways ^7^. The BLA also plays a crucial role in extinction of drug-seeking through its involvement in extinction memory consolidation and influence on related neural circuits ^5^. Extinction of conditioned fear requires neuronal activity in the BLA ^24^ and IL ^25^, as well as functional connectivity between the mPFC and amygdala ^26,27^.

However, how drug-seeking alters synaptic plasticity between the BLA and the IL remains unknown, and our LFP recordings are the first to directly examine synaptic changes in this pathway in-vivo. Our results show that both cocaine self-administration and cue-induced reinstatement paired with SHAM stimulation significantly strengthened baseline synaptic responses (measured as I-O curves) and enhanced LTP induction in the BLA-IL pathway. This suggests cocaine-seeking behavior hyperactivates this pathway to drive maladaptive cue-driven responses. How these changes in synaptic strength relate to the IL’s role in extinction learning remains unclear. Extinction paired with SHAM stimulation partially reduced cocaine self-administration effects as the amplitude of LTP in SHAM-stimulated rats following reinstatement was significantly smaller than in the COC group (Fig. 2H). Importantly, pairing extinction with VNS reversed both the increase in baseline responses in the BLA-IL projection and the amplification of LTP in this pathway that accompanied drug-seeking, resulting in only a small LTP resembling HFS-induced changes in synaptic plasticity seen in YS-control rats (Fig. 2H). Projections from the BLA to the mPFC contact both pyramidal projection neurons ^28^ and inhibitory interneurons ^29^. In the IL, interneuron activation drives robust feedforward inhibition ^29^, so the overall impact of the BLA projection may be predominantly inhibitory. Thus, increased strength and plasticity in the BLA-IL projection induced by cocaine self-administration and reinstatement may lead to IL inhibition and increased drug-seeking behavior ^4^. In contrast, extinction (alone or paired with VNS) may reduce this projection’s strength, leading to IL disinhibition and improved extinction memory expression. The reduction in synaptic strength in the BLA-IL pathway following VNS is noteworthy for another reason: Several studies show that VNS potentiates synaptic responses relative to control conditions ^17,30^ or enhances LTP ^31^. Our study provides the first evidence that pairing behavior with VNS can also lead to synaptic response weakening, even if other pathways’ strength was enhanced in the same subject. The mechanisms remain unclear, but they underscore the pathway-specificity of VNS modulation.

The NAc core regulates reward evaluation and motor activity. Drug-seeking is associated with increased excitability of PL-NAc core projecting neurons ^32^, and drug-induced changes in PL-NAc glutamate transmission drive drug-taking, relapse, and withdrawal. Repeated cocaine exposure strengthens glutamatergic transmission in the PL-NAc pathway ^2,33^, while PL inhibition can attenuate cue-induced reinstatement of cocaine seeking ^34^. Multiple lines of evidence support that chronic cocaine exposure induces LTP-like changes in nucleus accumbens medium spiny neurons ^2^, including increased AMPA/NMDA ratios of synaptic currents ^35,36^, upregulated membrane insertion of AMAP receptors into synaptic membranes ^37,38^, and heightened behavioral sensitivity to locally administered AMPA ^39,40^. Consistent with these findings, we show that cocaine self-administration and reinstatement significantly increased baseline eLFP amplitudes relative to YS group levels, suggesting drug-seeking strengthened the PL-NAc core pathway. High frequency stimulation induced persistent LTP in YS control animals, consistent with previous findings ^2^. In contrast, cocaine self-administration and reinstatement in SHAM-stimulated rats altered metaplasticity in this pathway, not only reducing LTP induction ability as previously shown ^2^, but causing LTD emergence. This suggests LTP induction in this pathway occurs on a sliding scale where prior drug exposure creates a condition where synapses are already potentiated (in an LTP-like state, evidenced by increased baseline responses in I-O curves), making further potentiation difficult due to ceiling effects, while depression remains inducible ^9^.

Similar drug-induced deficits in LTP or LTD induction in the NAc core have been shown for cocaine ^2,3^ or heroin ^41^. These synaptic changes likely underlie heightened sensitivity to drug-associated cues and increased relapse vulnerability ^42^. Importantly, we show that VNS modulates drug-induced alterations in synaptic plasticity within the PL to NAc core pathway. Pairing extinction with VNS reduced baseline eLFP amplitudes compared to recordings in COC and SHAM-treated rats, suggesting VNS reversed drug-induced changes in synaptic strength. VNS also partially reversed drug-induced metaplasticity changesseen in SHAM or COC groups, so that in the VNS group, HFS did not result in LTD. Together, these VNS-induced changes may attenuate pathological neuroplasticity associated with addiction, restoring the circuit’s capacity for bidirectional plasticity and countering the cocaine-induced “ceiling effect” that favors LTD over LTP. By normalizing exaggerated responsiveness to drug-associated cues, VNS might reduce the conditioned craving that drives relapse.

The IL plays a crucial role in the consolidation and expression of extinction memories ^43–45^, and it inhibits drug-seeking behavior through its projections to the NAc shell and/or the PL ^4,46,47^. Cocaine-seeking induced by IL inactivation is reversed by concurrent inactivation of the PL or BLA, suggesting that during extinction expression, IL actively competes with the cocaine-seeking circuitry ^4^. Extinction training induces plasticity in the IL-NAc shell projection ^48,49^, increasing AMPA receptor expression in the NA shell, with GluA1 levels negatively correlating with reinstatement susceptibility ^50^. Conversely, during drug seeking, activity in the IL-NAc shell connection may decrease ^32,51^, enabling the return of drug-seeking behavior following extinction. Our results provide further support for these ideas as rats in both COC and SHAM groups showed LTD, suggesting that drug-seeking weakened this pathway, consistent with previous studies demonstrating that IL inactivation induces cocaine-seeking ^44^. In contrast, VNS strengthened both the baseline response and increased metaplasticity in this pathway so that HFS of the IL caused pronounced LTP in the NAc shell. Together, these findings suggest drug-seeking behavior disrupts connections between the IL and NAc shell, whereas VNS strengthens the connection and enhances synaptic plasticity to aid extinction memory expression.

**Figure 5:**
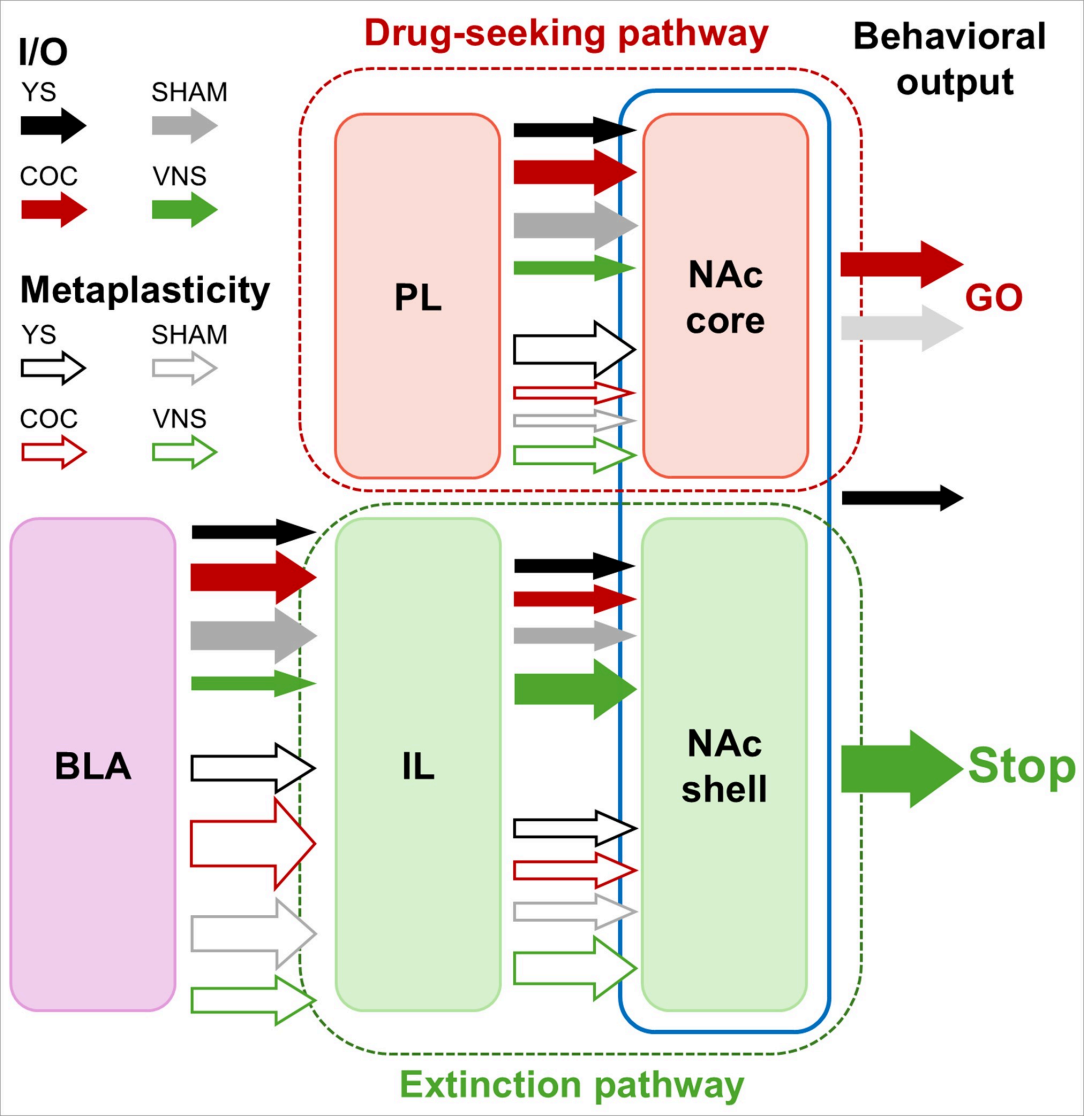
Drug-seeking and VNS change synaptic strength and metaplasticity across extinction and drug-seeking pathways. Summary of the effects of cocaine self-administration (COC) and extinction paired with either sham stimulation (SHAM) or VNS (VNS) followed by cue-induced reinstatement on baseline synaptic strength (filled arrows) and metaplasticity (open arrows) in three key pathways: BLA- IL, PL–NAc core (drug-seeking pathway), IL–NAc shell (extinction pathway). Arrows indicate relative changes in synaptic strength across treatments compared to yoked-saline (YS) groups, as well as changes relative to baseline in the case of synaptic plasticity. See text for details.

Activation of ascending vagus nerve fibers triggers release of various neuromodulators, including norepinephrine, acetylcholine, and BDNF, within the central nervous system, resulting in widespread cortical and subcortical activation ^52–57^. This release creates permissive conditions for synaptic plasticity, modulating cognitive and motivational states and influencing both sensory processing and encoding, as well as learning and memory-related processes ^17,58^. Specifically, long-term VNS increases norepinephrine and dopamine levels in the PFC and NAc, potentially influencing addiction-related neuroplasticity ^59^. VNS delivered during extinction from cocaine self-administration also increases BDNF levels in the mPFC, modulating glutamatergic transmission in the IL. Blocking TrkB receptors prevented both VNS’ effect on synaptic transmission and its beneficial effects on cue-induced relapse behavior ^60^. Consistent with our findings of VNS-induced network reorganization, studies in human patients with major depression indicate that VNS normalizes activity within the ventromedial prefrontal cortex, cingulate cortex, and limbic regions ^61^ and modulates pathways relevant for reward and motivation, strengthening the functional connectivity between the NAc, mPFC, and ACC ^62,63^. The VNS-induced changes in metaplasticity in the three pathways examined in our study similarly enhance capacity for adaptive plasticity in the corticolimbic network and contribute to VNS’ inhibitory effects on drug-seeking behavior (Fig. 5).

## Conclusion

Our study suggests that VNS modulates drug-seeking behavior by modulating drug-induced neuroplasticity within key corticolimbic circuits. VNS effectively enhanced extinction learning and suppressed drug-seeking behavior by reversing maladaptive synaptic changes induced by cocaine use and reinstatement. Specifically, VNS strengthened the IL-NAc shell pathway, crucial for extinction memory expression, and partially reversed synaptic plasticity in the PL-NAc core pathway that drives drug-seeking. These findings support the idea that VNS might serve as an adjunct to addiction treatment, offering a means for targeted synaptic plasticity to reduce relapse vulnerability.

## Materials and methods

### Animals and surgical procedures

Male and female Sprague Dawley rats (Envigo, ≥ 90 days old) were assigned to four groups: cocaine self-administration (COC, n = 16), yoked-saline control (YS, n = 14), and cocaine self-administration followed by extinction training with either sham-stimulation (SHAM, n = 16) or vagus nerve stimulation (VNS, n = 18). Rats were individually housed on a 12-hour reverse light/dark cycle with ad libitum food and water access. All protocols complied with the NIH Guide for Laboratory Animal Care and were approved by The University of Texas at Dallas IACUC committee and were conducted in accordance with the ARRIVE guidelines for reporting animal research. Rats were anesthetized with ketamine HCl (87.5 mg/kg) and xylazine (5 mg/kg) and implanted with jugular vein catheters and a custom cuff electrode around the left vagus nerve for VNS delivery ^14,60^. Catheters were flushed daily with gentamicin (2–3 mg/day/animal) and heparin (0.2 ml of 100 units) to prevent infection and maintain catheter patency. Ketoprofen was also administered to reduce pain and discomfort.

### Drug self-administration, extinction training, and VNS treatment

Drug self-administration and extinction training were performed as previously described ^14,60^. After 7–10 days of recovery, rats learned to self-administer food pellets in a single overnight session before beginning daily cocaine self-administration sessions in operant conditioning chambers (Med Associates). During the 2 hr. self-administration sessions, each active lever press delivered 0.25 mg cocaine (NIDA Drug Supply Program) in 0.05 ml saline over 3 seconds and the presentation of drug-paired cues (illumination of the light over the active lever and the presentation of a 2900 Hz tone), followed by a 20-second timeout. Subjects completed at least 10 days of self-administration with ≥ 20 infusions per session. Rats in the YS groups received saline infusions contingent on lever presses by cocaine-administering rats. Extinction groups underwent 10 days of training where lever presses no longer produced cocaine infusions or presentation of drug-paired cues. During extinction, rats received either sham stimulation or noncontingent VNS (0.4 mA for 30 seconds every 5 minutes). Following 10 days of extinction training, drug-seeking behavior was reinstated in a cue-induced reinstatement session during which presses on the active lever triggered the presentation of the previously drug-paired tone and light but did not result in drug delivery or VNS.

### In vivo LFP recording

Evoked local field potentials (eLFPs) were recorded after either the final self-administration session (COC, YS) or after cue-induced reinstatement (SHAM, VNS). Under urethane anesthesia (1.5 g/kg, i.p.) we recorded eLFPs in three pathways: For recordings of eLFPs in the BLA-IL pathway, a bipolar matrix stimulation electrode (FHC) was placed in the BLA (DV: 7.3, AP: −2.7, ML: 4.9 from bregma) and eLFPs were recorded in the IL (DV: 5, AP: +3, ML: 0.6 from bregma) using a tungsten microelectrode (WPI). For recordings in the IL-NAc shell pathway, the stimulation electrode was placed in the IL (DV: 5, AP: +3, ML: 0.6 from bregma), and eLFPs were recorded in the NAc shell (DV: 7.3, AP: +1.5, ML: 0.8 from bregma). Field potentials in the BLA-IL and IL-NAc shell pathways were recorded in opposing hemispheres in the same rats. We alternated both hemispheres and the order of the recordings across all animals. For recordings of eLFPs in the PL-NAc core pathway, the stimulation electrode was placed in the PL (DV: 3.5, AP: +3, ML: 0.6 from bregma) and recordings were performed in the NAc core (DV: 7, AP: +1.5, ML: 1.4 from bregma). Field responses were evoked every 15 s using a 0.3 ms stimulation pulse, and the basal stimulation intensity corresponded to 40% of the minimum current intensity that evoked a maximum field response, based on an input–output curve determined before collection of baseline data. Signals were amplified using a Model 1600 Neuropore Amplifier (A-M Systems) and a BMA 200 Portable Bioamplifier (CWE, cwe-inc.com). Signals were digitized using a CED 1401 interface (Cambridge Electronic Design, Cambridge, England) and analyzed using Spike-2 (CED). Baseline data were collected for a minimum of 10 min before synaptic plasticity was induced using high-frequency stimulation (HFS) consisting of three bursts of 100 pulses at 50 Hz (2 s), with 20 s inter-burst intervals at the minimum current intensity that evoked the maximum field response. Field potential amplitude was measured as the difference between the mean of a 5 ms window before the stimulation artifact and the negative peak of the field potential after the stimulation artifact. Responses were averaged across 2 min for analysis. Data were normalized to baseline, and the average of a 10 min baseline was set as 100%, and the 10 min period 40–50 min after plasticity induction was used to analyze long-term synaptic changes.

### Data analysis

Statistical analyses used GraphPad Prism 7.0.5. Active lever presses during self-administration and extinction were compared using separate two-way ANOVAs with the factors of treatment and time following with Post hoc analyses. An independent t-test was conducted on the cue-induced reinstatement session to compare reinstatement between VNS and SHAM animals. Changes in eLFP amplitudes following induction of synaptic plasticity were analyzed using repeated-measures ANOVA with a treatment group × time interaction. Input-output curves of eLFPs were analyzed using two-way repeated measures ANOVA with the factors of treatment group × current intensity. *P* values < 0.05 were considered significant.

## Data Availability

The data that support the findings of this study are available from the corresponding author upon reasonable request.
